# Supplementary material for: Deletion of wfs1 Impairs Oligodendrocyte Precursor Cells Dorsal Distribution and Myelination Through the wfs1-hmgcs1 Axis in Zebrafish
Source: Biology (Basel). 2026 Aug 16;15(16):1402. doi: 10.3390/biology15161402 (PMC13510151; doi:10.3390/biology15161402)
Supplement: Supplementary file 1 [file biology-15-01402-s001.zip › File S1-Transcriptome sequencing data and differentially expressed genes (DEGs) in wfs1-- zebrafish at 5 dpf/1.GO/wfs1KOvsWT/GSEA/WT/MIDBODY/MIDBODY(GO_0030496).html]

Details for gene set MIDBODY(GO:0030496)[GSEA]


|  || Dataset | deg.sample |
| Phenotype | sample.cls |
| Upregulated in class | WT |
| GeneSet | MIDBODY(GO:0030496) |
| Enrichment Score (ES) | -0.4989567 |
| Normalized Enrichment Score (NES) | -1.9180998 |
| Nominal p-value | 0.0 |
| FDR q-value | 0.18190354 |
| FWER p-Value | 0.352 |
Table: GSEA Results Summary

  

Fig 1: Enrichment plot: MIDBODY(GO:0030496)
  
Profile of the Running ES Score & Positions of GeneSet Members on the Rank Ordered List

  

| PROBE | DESCRIPTION   (from dataset) | GENE SYMBOL | GENE\_TITLE | RANK IN GENE LIST | RANK METRIC SCORE | RUNNING ES | CORE ENRICHMENT || 1 | 567134 | zgc:153916 |  |  | 4472 | 0.749 | -0.1074 | No |
| 2 | 101883254 | si:ch1073-266p11.2 |  |  | 4699 | 0.719 | -0.0829 | No |
| 3 | 553631 | katna1 |  |  | 5234 | 0.652 | -0.0709 | No |
| 4 | 323182 | pkn3 |  |  | 6375 | 0.549 | -0.0825 | No |
| 5 | 573631 | rab11fip3 |  |  | 6523 | 0.535 | -0.0636 | No |
| 6 | 492353 | ddx11 |  |  | 6808 | 0.510 | -0.0500 | No |
| 7 | 571965 | pkn2 |  |  | 8676 | 0.389 | -0.0915 | No |
| 8 | 394136 | sept7a |  |  | 9042 | 0.365 | -0.0869 | No |
| 9 | 436851 | ankrd54 |  |  | 9735 | 0.319 | -0.0946 | No |
| 10 | 436685 | zfyve19 |  |  | 10603 | 0.266 | -0.1101 | No |
| 11 | 791894 | aurkb |  |  | 11616 | 0.210 | -0.1326 | No |
| 12 | 445479 | agbl5 |  |  | 12023 | 0.189 | -0.1370 | No |
| 13 | 322309 | sirt2 |  |  | 13923 | 0.096 | -0.1924 | No |
| 14 | 553475 | alkbh4 |  |  | 14656 | 0.062 | -0.2126 | No |
| 15 | 323457 | sept2 |  |  | 14860 | 0.053 | -0.2167 | No |
| 16 | 797451 | arl3 |  |  | 15068 | 0.043 | -0.2213 | No |
| 17 | 393170 | mitd1 |  |  | 15085 | 0.042 | -0.2200 | No |
| 18 | 767769 | cep44 |  |  | 16054 | -0.003 | -0.2502 | No |
| 19 | 565434 | usp8 |  |  | 17506 | -0.054 | -0.2934 | No |
| 20 | 560832 | jtb |  |  | 18345 | -0.092 | -0.3157 | No |
| 21 | 794166 | ccdc69 |  |  | 18766 | -0.112 | -0.3239 | No |
| 22 | 405851 | spast |  |  | 19339 | -0.137 | -0.3359 | No |
| 23 | 393164 | chmp4ba |  |  | 19448 | -0.142 | -0.3330 | No |
| 24 | 100000597 | sept7b |  |  | 20105 | -0.171 | -0.3461 | No |
| 25 | 336839 | cdc42 |  |  | 20515 | -0.189 | -0.3506 | No |
| 26 | 393431 | zgc:63587 |  |  | 20871 | -0.208 | -0.3526 | No |
| 27 | 436806 | rab11fip4a |  |  | 20998 | -0.215 | -0.3470 | No |
| 28 | 570926 | lzts2a |  |  | 22187 | -0.278 | -0.3721 | No |
| 29 | 553800 | cep55l |  |  | 22351 | -0.287 | -0.3646 | No |
| 30 | 100005471 | lzts2b |  |  | 23135 | -0.336 | -0.3744 | No |
| 31 | 100037321 | zgc:162255 |  |  | 25227 | -0.462 | -0.4197 | No |
| 32 | 393537 | ccdc124 |  |  | 25491 | -0.483 | -0.4066 | No |
| 33 | 324565 | zfyve26 |  |  | 25745 | -0.507 | -0.3923 | No |
| 34 | 100003958 | si:dkey-3k24.5 |  |  | 26366 | -0.563 | -0.3869 | No |
| 35 | 561486 | cep126 |  |  | 27011 | -0.630 | -0.3794 | No |
| 36 | 100151129 | rab11fip4b |  |  | 30821 | -1.778 | -0.4206 | Yes |
| 37 | 792259 | sept15 |  |  | 31355 | -2.543 | -0.3254 | Yes |
| 38 | 557239 | antxr2a |  |  | 31582 | -3.209 | -0.1911 | Yes |
| 39 | 793158 | cdc42l |  |  | 31794 | -4.556 | 0.0030 | Yes |
Table: GSEA details
[plain text format]

  

Fig 2: MIDBODY(GO:0030496)
  
Blue-Pink O' Gram in the Space of the Analyzed GeneSet

  

Fig 3: MIDBODY(GO:0030496): Random ES distribution
  
Gene set null distribution of ES for
 **MIDBODY(GO:0030496)**
